# Synchronization Modulation Increases Transepithelial Potentials in MDCK Monolayers through Na/K Pumps

**DOI:** 10.1371/journal.pone.0061509

**Published:** 2013-04-09

**Authors:** Vu Tran, Xiaodong Zhang, Lin Cao, Hanqing Li, Benjamin Lee, Michelle So, Yaohui Sun, Wei Chen, Min Zhao

**Affiliations:** 1 Institute for Regenerative Cures, Departments of Dermatology and Ophthalmology, University of California Davis, Davis, California, United States of America; 2 Cellular and Molecular Biophysics, Department of Physics, University of South Florida, Tampa, Florida, United States of America; 3 Center for Neurosciences, University of California Davis, Davis, California, United States of America; University of Texas Health Science Center at Tyler, United States of America

## Abstract

Transepithelial potential (TEP) is the voltage across a polarized epithelium. In epithelia that have active transport functions, the force for transmembrane flux of an ion is dictated by the electrochemical gradient in which TEP plays an essential role. In epithelial injury, disruption of the epithelial barrier collapses the TEP at the wound edge, resulting in the establishment of an endogenous wound electric field (∼100 mV/mm) that is directed towards the center of the wound. This endogenous electric field is implicated to enhance wound healing by guiding cell migration. We thus seek techniques to enhance the TEP, which may increase the wound electric fields and enhance wound healing. We report a novel technique, termed synchronization modulation (SM) using a train of electric pulses to synchronize the Na/K pump activity, and then modulating the pumping cycles to increase the efficiency of the Na/K pumps. Kidney epithelial monolayers (MDCK cells) maintain a stable TEP and transepithelial resistance (TER). SM significantly increased TEP over four fold. Either ouabain or digoxin, which block Na/K pump, abolished SM-induced TEP increases. In addition to the pump activity, basolateral distribution of Na/K pumps is essential for an increase in TEP. Our study for the first time developed an electrical approach to significantly increase the TEP. This technique targeting the Na/K pump may be used to modulate TEP, and may have implication in wound healing and in diseases where TEP needs to be modulated.

## Introduction

### Polarized epithelia maintain voltage differences between apical and basal sides, which are termed transepithelial potentials (TEP)

TEP represents energy consuming transport/fluxes of ions across the epithelium. In a monolayer, the sum of the membrane potentials for the basal and apical cell membranes is the TEP. TEPs have physiologically important functions. The force for the transmembrane flux of ions is dictated by the electrochemical gradient. In transporting epithelia, such as in the kidney and intestines, the TEPs facilitate transport of ions, nutrients and metabolites. In the kidney, TEPs of 2–3 mV contribute to tubular reabsorption [1]–[3]. Multilayered epithelia also maintain TEPs. The corneal and skin epithelia maintain TEPs about 25–40 mV, basal side positive relative to the apical side [4]–[9]. The thickness of the epithelial layers is about 50–70 µm, thus forming field strength up to 500 mV – 1000 mV/mm.

### One possible function of TEPs is to signal injury when the epithelial barrier is compromised and enhance wound healing

A wound disrupts the epithelium, breaks the epithelial barrier and generates laterally orientated wound electric fields. The positive potentials drive electric current flows to wound edges where the TEP collapses. This laterally orientated electric field measures about ∼40 mV/mm or more [10]–[17]. The naturally occurring wound electric fields guide epithelial cells to migrate directionally towards the cathode, the direction of the wound center. Being able to enhance the TEPs may help to modulate transepithelial transport, or to increase wound electric fields, and ultimately wound healing. We have previously used pharmacological agents to increase TEP in the cornea up to 30% and significantly increased wound-healing rate [18].

### How the TEPs are generated is not fully understood

In the cornea, collective transport of Na+ and Cl- is mainly responsible for TEP [7], [11], [18]–[20]. In both monolayer epithelia and stratified epithelia, the basal side has a higher expression level of ATPases than the apical side. The ATP pumps are asymmetrically distributed to the basal side [21], [22]. In skin epithelium, Na/K pump expression in an asymmetric fashion is followed by the establishment of a TEP, which is sensitive to Na channel inhibitors such as ameloride [8]. In kidney epithelial cells, there is also a high density of the Na/K pump molecules located in the basolateral membrane. The pump molecule extrudes three Na ions and intrudes two potassium ions by consuming the energy from hydrolysis of one ATP molecule in each pumping cycle. As a result, a high concentration gradient of Na ions is built up across the cell membrane. Various kinds of Na-cotransporters located on the apical side of the epithelium consume the energy stored in the electrochemical potential of Na concentration gradient to transport various ions. As a result, net cation transport or ion flux occurs across the epithelial cells showing a potential difference (TEP) across the epithelium.

### Manipulation of the TEPs may affect many functions of epithelia

Limited methods are available to enhance TEPs. We used pharmacological agents to increase transcorneal potential differences, in which TEPs account for nearly 70–80% [18]. Recently, we developed a novel technique so called synchronization modulation (SM) to physically control and manipulate the function of the Na/K pumps [23]–[27]. The underlying mechanisms involved in the technique have been reported previously [28]. Briefly, the concept was introduced from an electronic synchrotron accelerator. First, a specially designed oscillating electric field is used to synchronize all the pump molecules to run at the same pace as the applied electric field. This is achieved by entrapping the pumps' two limbs, Na- and K-transports, into the positive and negative half-cycle of the oscillating electric field, respectively. Then, by gradually changing the field oscillating frequency, these pump molecules can be entrained to a defined value of the pumping rate. The technique has been successfully applied to skeletal muscle fibers [27], [29], cardiomyocyte [26], [30], cultured cell line, blood vessels, and kidney proximal tubules. The results showed that the synchronization modulation technique can effectively activate the pumping rate up to ten fold quickly in tens of seconds.

We also used this novel technology - synchronization modulation stimulation (SM) to target and enhance the function of Na/K pumps in cultured monolayer of epithelium. SM stimulation could successfully increase membrane potentials in neurons and cardiomyocytes. In this report, we for the first time demonstrate that SM significantly enhances TEPs in MDCK epithelial monolayer by three to four fold. This increase is mediated by the Na/K pump and its asymmetric expression along the MDCK monolayer. SM therefore is able to facilitate effective transport of the Na/K pump and increase TEPs. This method may find important clinical uses where TEPs need to be increased.

## Materials and Methods

### Reagents and cell culture

**Figure 1:**
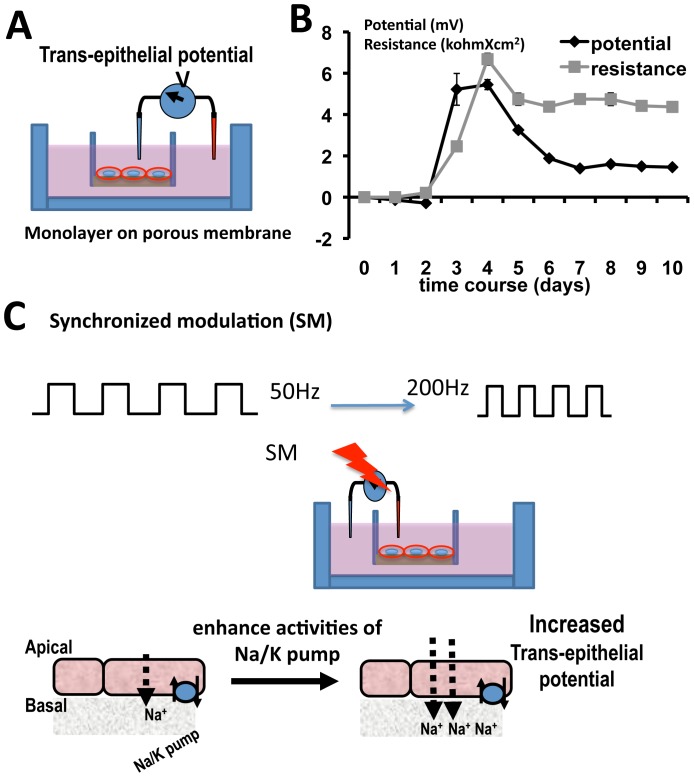
Schematic diagram of synchronization modulation (SM) on transepithelial potentials (TEP). **A.** TEP measurements with the insert culture. **B.** MDCK cells in the insert culture establish TEP and transepithelial resistance (TER), which stabilized ∼6 days in our culture system. Time course of typical TEP and TER. A total of 22 Millipore wells containing MDCK Type 1 cells were monitored each day for 10 days. The TEP and TER are superimposed for comparison. Both recordings peaked at ∼4 days then decreased to stable resting values from day 6–7 onward. An intact monolayer formed at day 3 as the TER started to increase. **C.** SM forces the Na/K pumps to work at higher rates and causes a greater net flow of Na (positive charges) to the basal side. In a polarized epithelium where the Na/K pump is localized almost exclusively on the basolateral membrane, SM results in increases in the transepithelial potential. Fig. 1B n = 22 for all data points.

MDCK cells were grown in DMEM/F12 medium supplemented with fetal bovine serum (FBS), non-essential amino acids, and antibiotic/antimycotic solution. Cell growth medium was changed every two to three days. Once 70% to 80% confluent, the cells were sub-cultured for at least 5 passages before they were seeded onto Millicell® 24-well cell culture insert plates. The insert plates have a 0.4 µM pore base of polycarbonate membranes on which cells can grow and form tight monolayer separating the apical and basal sides (Sigma) (Fig. 1A).

### Transepithelial potential (TEP) and transepithelial resistance (TER) measurements

We used a Millicell®-ERS Electrical Resistance System voltohmeter (Millipore Corporation) to monitor the resistance of MDCK monolayers following manufacturer's instruction (Fig. 1A). The same device was then used to record the TEP before and after application of the synchronization modulation electric field. Each time, two medium-loaded wells with no cells were used to standardize (zero) the voltohmeter before each recording. We only used wells that established stable TEP and TER for the stimulation experiments. About 4 days after culture, the TER reached 3–5 KΩ×cm^2^ and a resting TEP reached 0.5–1 mV, which we selected for stimulation experiments. In a few cases the monolayers were damaged, which caused leakage and drop of TER and TEP. These wells were excluded from further experiments.

### Na/K Pump inhibition

Two pump inhibitors, ouabain and digoxin, were used to inhibit the Na/K pump. Both drugs bind and inhibit Na/K pump activity. Ouabain was used in concentrations of 1 µM to 1 mM. Digoxin, another potent inhibitor of the Na/K pump was added in concentrations of 0.1 µM to 10 µM. The cells were treated with the medium containing drugs as indicated for 1 hr. To exclude long-term non-specific cytotoxic effects, the drugs were used for a short period of time upon experimentation.

### Disruption of asymmetric expression of the Na/K pump

Butyrate was used to disrupt asymmetric expression of the Na/K pump to the basolateral side. Butyrate was added at 5 mM for 24 hrs. All drugs were present in the culture medium and applied at both the apical and basolateral side.

### Synchronization-modulation stimulation

We used a patented technique – synchronization modulation (SM) stimulation to modulate TEPs. A custom-made signal generator (Chen) was used to provide the synchronization modulation electric field. The parameters of the electric field were reported previously [25]. The device has three modes: forward modulation, backward modulation, and no modulation. In this study, we applied the synchronization modulation electric field in all three modes. In forward modulation mode the electric field oscillated initially at a frequency of 50 Hz for 5 s. Then, the field frequency was gradually increased to 200 Hz in a stepwise pattern within 30 s. This mode works to accelerate the pump molecules from their natural turnover rate, 50 Hz, to 200 Hz. In order to prove the field-induced effect is due to the pre-programmed synchronization modulation, we applied the electric field for the same magnitude and period in the backward modulation mode starting from a frequency of 50 Hz and ending at 10 Hz, and in no modulation mode where the oscillating frequency remains at a constant frequency of 50 Hz. The electric fields were all delivered at a constant 2 V for 30 s to our MDCK monolayers and applied individually to each well one at a time. The waveforms were confirmed with an oscilloscope. Synchronized modulation was applied at 7 days after seeding MDCK cells onto the culture insert plates.

The stimulation was applied with two Ag/AgCl electrodes. The wire tips were bathed in medium across the monolayer in the insert culture and arranged in an apical to basolateral fashion (Fig. 1C). Cells cultured in the Millipore® wells shared a single feeder tray when first seeded but were changed to separate feeder trays, where each insert had its separate containing well one day before experimentation.

### Microscopy

One-week culture of MDCK monolayer was fixed in 4% paraformaldehyde for 20 minutes. Cells were permeabilized (5 mins) and blocked with 10% normal serum from the same species as the secondary antibody, 1% BSA and 0.02% NaN_3_ in PBS for 30 minutes, followed by 2 hours incubation with Na^+^/K^+^ ATPase (Abcam) antibodies. After washing with PBS, cells were incubated with Alexa Fluor® highly cross-adsorbed secondary antibodies (Invitrogen) for 1 hour. Cells were washed three times, mounted in Vector mounting medium with DAPI. Images were visualized on a Zeiss LSM 510 META Confocal microscope using 20X/0.5w and 40X/1.2w objectives.

### Statistical analysis

All statistical tests were computed using Excel 2008. Data are given as means±standard error mean. The Student's two-sample *t*-test was used to compare relevant differences between treatment groups and controls. *P*-values <0.05 are indicated for those differences.

## Results

### MDCK monolayers established stable TEP and TER

When MDCK form monolayer, Na/K pumps distribute to the basolateral side of the polarized epithelium. The activities of Na/K pumps are electrogenic because it exchanges 3Na^+^ for 2K^+^ with every molecule of ATP used. Each pumping cycle results in a net flow of one positive charge to the outside of the cell, i.e. to the basolateral side.

We first established stable base conditions of TEP and TER. The TEP and TER of epithelial monolayer in Millipore wells were monitored each day for up to 10 days. Two days after seeding the cells, the measurements showed gradual increases of TER and TEP, indicating tight junction formation, which limits free movement of ions across the monolayer. This intact monolayer formation corresponded to when the TEP started to increase. Both TER and TEP reached peaks on day 4, then decreased and maintained consistent values without much variation between experiments from day 6∼7 onward (Fig. 1B).

The rational to enhance TEP is that by synchronization modulation of the Na/K pump located in the basolateral membrane of the epithelium, the pumping rate was accelerated from the natural turnover rate of 50 Hz to 200 Hz. Each pump molecule pumps out one net cation in one pumping cycle. Consequently, the Na concentration gradient across the cell membrane is significantly increased, or the intracellular potential becomes more negative. This electrochemical potential will enhance various Na-cotransporters on the apical membrane to transport Na ions into the cell at the apical side. As a result, an increased net cation flux across the epithelium occurs representing an increased transepithelial potential difference. Because of the asymmetric expression of Na/K pumps at the basolateral side, increased frequency of transport means more Na would be pumped to the basal side, increasing the TEP (Fig. 1C).

### Synchronization modulation significantly increased TEP

**Figure 2:**
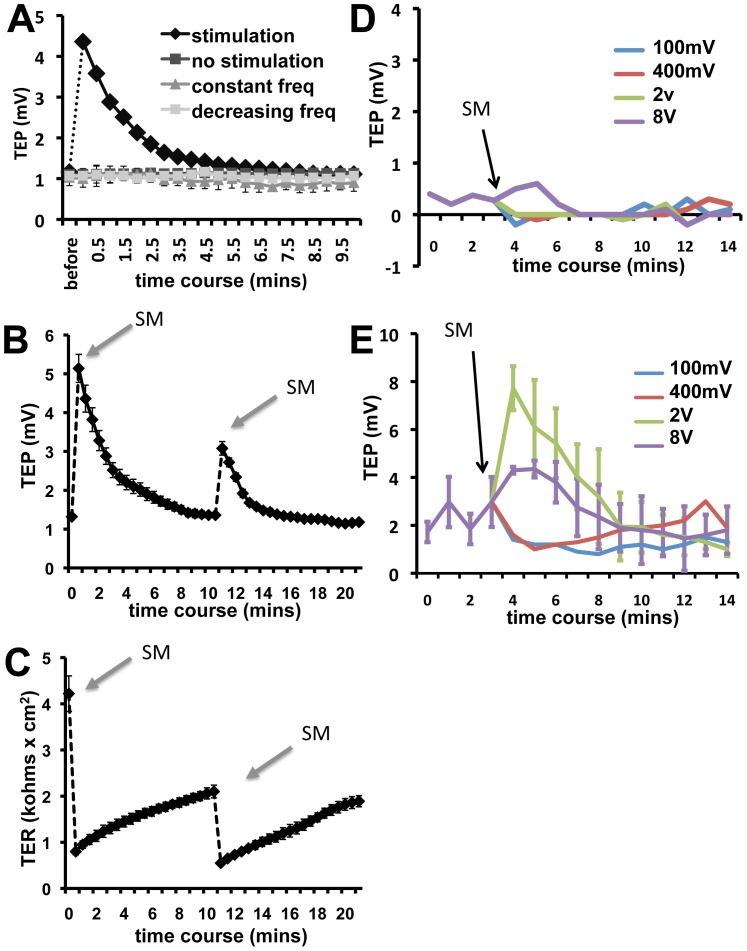
Synchronization modulation significantly increased TEP. **A.** TEP increased by more than four-fold following SM stimulation (2 V for 30 secs). TEP returned to resting values in∼10 mins. Setting the stimulator to elicit a constant or decreasing oscillation frequency showed no TEP response. **B.** TEP increase can be repeatedly induced by SM stimulation, albeit with decreased magnitude. **C.** SM caused drops in TER, which steadily recovered to less than half of the resting values before SM stimulation. Both applications of the stimulator reduced TER to less than 1 KΩ×cm^2^. **D**. Monolayer cultures of CHO cells, which do not form tight junction and thus are leaking were used as a control. **E.** Comparison of different voltages used for SM. Fig. 2A, D, & E n > 3 for all data points, Fig. 2B & C n = 5.

We next used SM stimulation to enhance the TEP. Indeed, SM stimulation significantly increased the TEP of MDCK monolayer (Fig. 2A). In our preliminary experiments, we tested four voltages, 100 mV, 400 mV, 2 V, and 8 V (Fig. 2E). Stimulation using 2 V increased TEP the most. We thus chose 2 V for all following experiments reported unless otherwise indicated. SM stimulation increased the TEP by more than four-fold from 1 mV to close to 5 mV (Fig. 2A). Stimulation of 30 secs at 2 V resulted in a sharp increase to peak TEP. This increase was abolished and recorded with the voltohmeter as soon as we removed the SM stimulator. Although there were variations in stimulation responses from the baseline of 1 mV – 2 mV to 4.5 mV – 7.5 mV, respectively, cells with TEP at either baseline showed very consistent 3–4 fold increase (Fig. 2A & E).

To confirm that this enhancement effect is due specifically to the special stimulation SM scheme, we tested two other stimulation schemes. The first scheme entails setting the stimulator to apply a constant oscillation frequency of 50 Hz instead of the 50 Hz to 200 Hz modulation at the same voltage for the same period of time. The second scheme is setting the stimulator to a decreasing oscillation frequency from 50 Hz to 10 Hz. Neither of these stimulation schemes elicited any changes in the TEP (Fig. 2A).

Switching off stimulation resulted in a gradual decrease of the TEP, which stabilized back to the basal line within 5–10 min. Repeating the stimulation elicited another increase in TEP with a smaller magnitude (Fig. 2B). Synchronized modulation caused a sharp drop in TER, which steadily recovered to resting values. The stimulation reduced TER to less than 1 KΩ×cm^2^, down from about 4.2 KΩ×cm^2^ the first time, and from 2.1 KΩ×cm^2^ the second time (Fig. 2C).

We also used Chinese Hamster Ovary (CHO) cells as a control, which do not form monolayer with tight junction. SM stimulation with voltages of 100 mV, 400 mV, 2 V, 8 V did not affect the transwell measurements of TEP (Fig. 2D).

MDCK TEP easily responded to re-stimulation at 10 min but the rise was only about half its original value (around 3 mV compared to an initial 5 mV). This reduced increase in TEP could be explained by a decline in transepithelial resistance (TER). Fig. 2C indicates that TER dropped from over 4 KΩ×cm^2^ to under 1 KΩ×cm^2^ during the 30 sec in which synchronized modulation was applied. Although TER steadily increased after removal of the stimulator, it recovered to only about 2 KΩ×cm^2^ when synchronization modulation was reapplied. The weaker increase in TEP thus parallels the lower starting TER upon re-stimulation. Interestingly, stimulation of the same samples 3 hrs later showed full increases in TEP. This response most likely coincides with full recovery of TER to around 4 KΩ×cm^2^. Experiments on different MDCK cell lines further support the importance of TER on TEP response. We cultured and stimulated both high resistance MDCK Type I cells and low resistance MDCK Type II cells. Measurements after 10 days of incubation showed that the TER of MDCK Type I cells stabilized at around 4 KΩ×cm^2^ while the TER of MDCK Type II cells remained much less than 1 KΩ×cm^2^, both of which corresponds well to published literature [31]–[33]. Concurrent stimulation led to immediate increases in TEP for MDCK Type I cells but no observable changes with Type II cells. We hypothesize that the low resistance of MDCK Type II corresponds to a dynamic leak that quickly diminished any electrical gradient that may have accumulated from stimulation. Conversely, data from MDCK Type I samples may have shown an even more enhanced and prolonged increase in TEP if TER did not decrease with synchronized modulation.

### SM induced increase in TEPs is mediated by the Na/K pump

**Figure 3:**
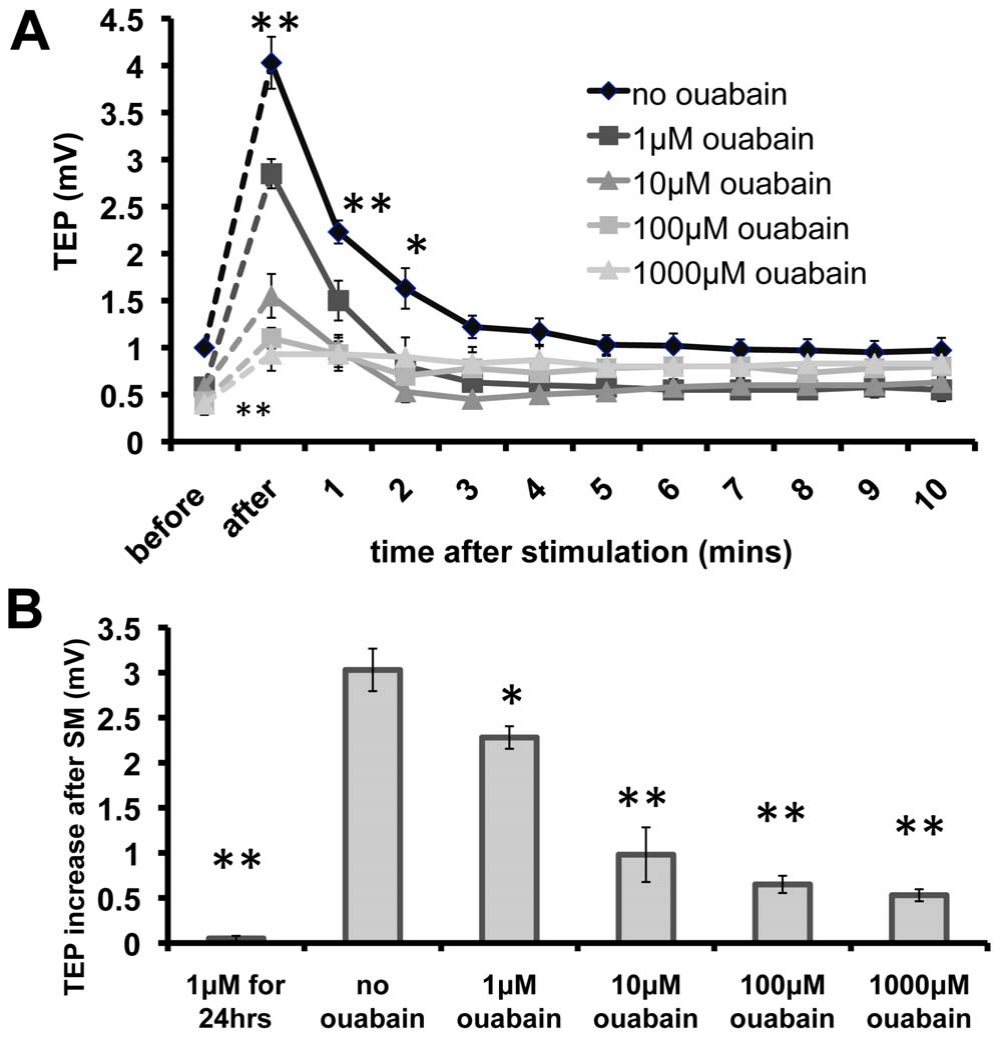
Synchronization modulation of TEP is mediated by the Na/K pump. **A.** Ouabain at all concentrations applied significantly decreased resting TEP, which was measured immediately after SM. Time courses depicting stimulation with and without ouabain showed that ouabain in 1 µM to 1000 µM progressively depressed TEP increases. **B.** Stimulation without ouabain increased TEP by about 3.03 mV from pre-SM values. This increase was significantly inhibited by ouabain treatment for 1 hr, suggesting that depolarization is offset by decreased activity of the Na/K pump. Stimulation applied to cells incubated with 1 µM ouabain for>24 hrs elicited almost no response. * p<0.05, ** p<0.01, all comparing no ouabain control with ouabain treated monolayers (A: top line vs. all other lines, or B: second column vs. all other columns). N  =  4–6 for all data points.

To investigate the molecular targets of SM, we used ouabain to block the Na/K pump. Ouabain treatment abolished the effects of SM on TEP (Fig. 3). Ouabain at all concentrations significantly decreased the resting transepithelial potentials of MDCK monolayers. Ouabain treatment for 1 hr decreased the resting potential from about 1 mV to 0.5 mV. Stimulation of control MDCK monolayers without the presence of ouabain increased transepithelial potentials by about 3.03 mV, compared to only 0.53 mV with 1 mM ouabain, suggesting that SM induced increase in TEP is offset by inhibited activity of the Na/K pump. The inhibition effect of ouabain is dose-dependent. Ouabain from 1 µM to 1000 µM treatment for about 1 hr each progressively decreased the amplitude of the SM-induced increases in TEPs. Stimulation applied to cells incubated with 1 µM ouabain for 2 days elicited no response (Fig. 3B).

To confirm the role of Na/K pumps, we used another potent inhibitor of the Na/K pump, digoxin. Digoxin also suppressed increases in TEP in a dose-dependent fashion. Digoxin at 0.1 µM showed little effect on SM-induced increase in TEP. Higher concentration of digoxin (10 µM) almost completely abolished SM-induced increase of the TEP.

**Figure 4:**
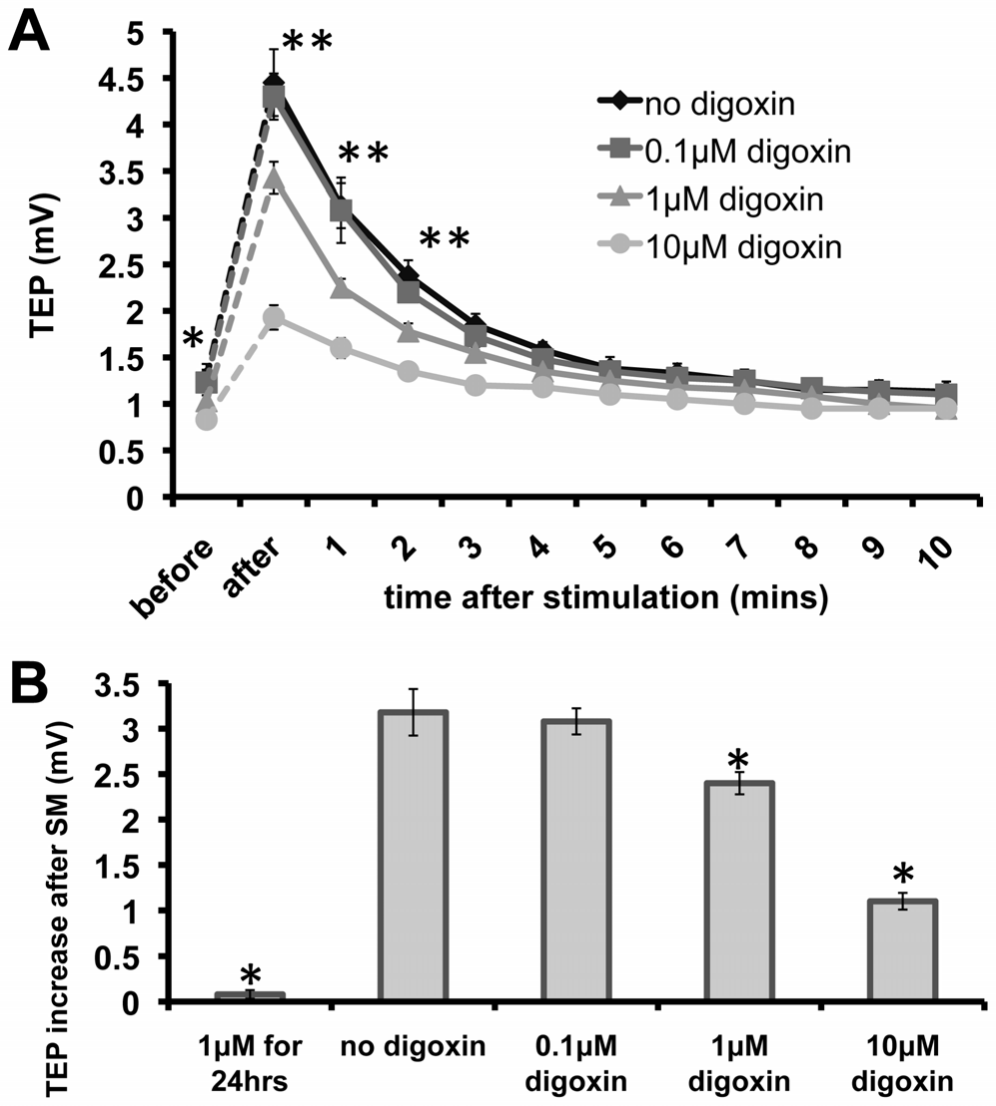
Digoxin decreased depolarization similar to ouabain. Application of digoxin, another inhibitor of the Na/K pump, also suppressed increases in TEP in a dose-dependent fashion. * p<0.05, ** p<0.01 when compared with no digoxin treated value (A: top line vs. all other lines, or B: second column vs. all other columns). N  =  4 for all data points.

To exclude possible non-specific cytotoxic effects of long-term treatment with ouabain and digoxin, MDCK cells were treated with 1 µM ouabain or digoxin for only 1 hr before SM stimulation. Both TEP/TER were still present after 1 hr treatment of ouabain or digoxin, but nonetheless, SM induced increase in TEP was significantly decreased (Figs. 3 and 4). We also exchanged the medium with drug-free medium after stimulation and cultured the cells in CO_2_ incubator for another two days. Re-stimulation of those cells showed normal TEP responses to stimulation after recovery of the resting TEP and TER (data not shown).

### Asymmetric distribution of the Na/K pump is required for SM induced increase of TEPs

**Figure 5:**
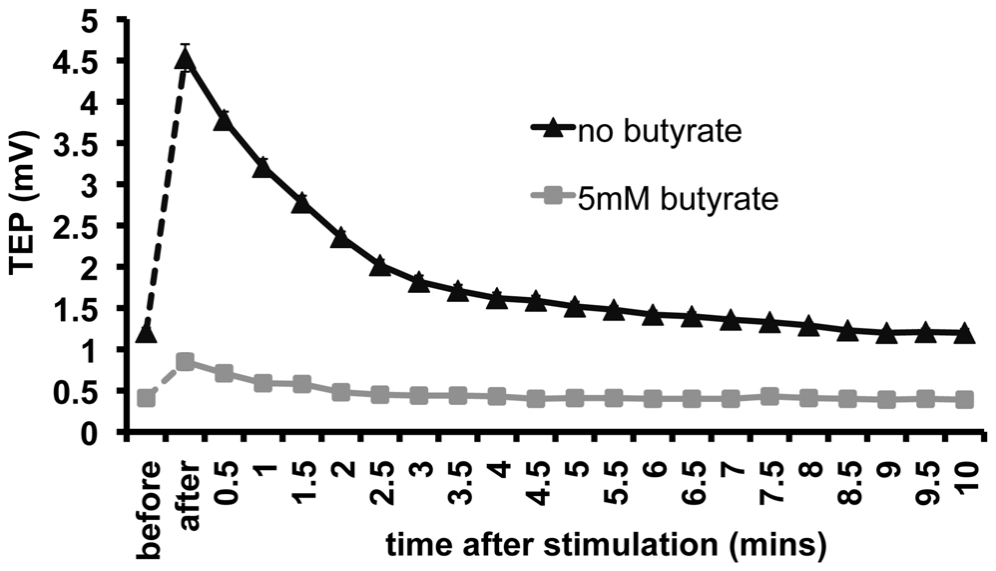
Asymmetric distribution of Na/K pumps is required for synchronization modulation of TEP. Butyrate, a short chain fatty acid known to disrupt the basolateral localization of Na/K pumps in MDCK cells, was added to the growth medium. Cells treated with butyrate only slightly depolarized when stimulated after 24 hrs whereas cells without butyrate dramatically depolarized upon stimulation as expected. After means immediately after stimulation. P<0.01 and n  =  5–8 for all data points.

To test the role of spatially polarized expression of Na/K pump on SM-induced increase of TEP, we used butyrate, a short chain fatty acid known to disrupt the basolateral localization of the Na/K pumps in MDCK cells. Butyrate was added to the growth medium. Incubation of cells with 5 mM butyrate for one day showed observable, but significantly decreased TEP and TER (from an average of 1 mV to 0.5 mV and of 4 KΩ×cm^2^ to 2.7 KΩ×cm^2^, respectively). After 24 hrs of treatment with butyrate, the monolayer showed only a slight increase in TEP upon SM stimulation, whereas control cells without butyrate treatment showed significant increase in the TEP (Fig. 5). Some cells were initially stimulated after 1 hr incubation with butyrate, but showed similar responses to control cells and were re-incubated (data not shown).

**Figure 6:**
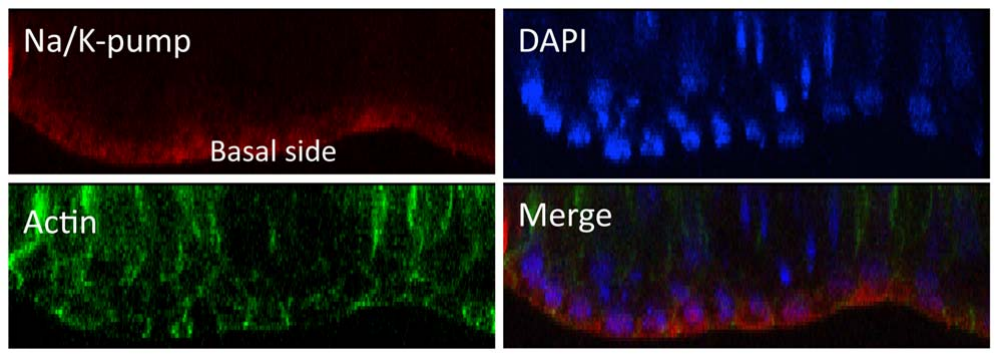
Na/K pump staining in MDCK monolayer. Specific immunohistochemistry staining localized Na/K pump expression to the basal side.

To confirm polarized expression of the Na/K pump we used specific immunohistochemical staining on MDCK monolayers. As predicted, the pumps localized to the basal side (Fig. 6).

### Differential TEP response to stimulation after prolonged pharmacological treatment supports their underlying mechanisms

Mechanistically, ouabain and digoxin function different to butyrate. The cardiac glycosides directly and reversibly inactivate the Na/K pump [34], [35] while butyrate induces irreversible cellular changes, leading to alterations in protein expression, cell cycle arrest, and eventually, apoptosis [36]–[38]. Loss of TER is also seen with prolonged butyrate treatment. This is due to butyrate-induced downregulation of the Na/K pump β1 subunit, which is responsible for mediating normal MDCK tight junction adhesion [39], [40]. As predicted, our cells similarly responded with limited TEP increases upon stimulation and elicited progressive TER recovery after addition of either 1 µM ouabain or digoxin for 1 hr, or 5 mM butyrate for 24 hrs (Figs. 3, 4 & 5). Replacing the cells with drug-free medium and then incubating them for another 24 hrs, however, reveal contrasting results. Re-stimulation of MDCK cells originally incubated with ouabain or digoxin showed normal responses but re-stimulation of cells originally incubated with butyrate showed no response, as the TEP and TER were both absent. While the glycosides can also induce apoptosis, MDCK viability and response to stimulation can be recovered with prompt change to drug-free medium. Butyrate, however, is noted to produce lasting changes even when removed. Importantly, our results show that synchronized modulation is a biological phenomenon and works only on active living cells.

## Discussion

TEPs have important functions, including absorption and secretion. TEPs may help to signal injury and facilitate wound healing, because the laterally orientated wound electric fields provide a powerful signal to stimulate and guide migration of epithelial cells in wound healing [12], [16], [17], [41]–[44]. Techniques that enable effective regulation of TEPs may be used as an experimental tool to study molecular mechanisms of TEP regulation, and to have potential clinical use where TEPs need to be regulated. In this report, we successfully developed a novel technique we named Synchronization Modulation (SM) to enhance TEPs over 4 fold. This is for the first time an electrical method has been used to increase the TEP. Furthermore, we demonstrated that the molecular target of the SM stimulation is the Na/K pump.

### Establishment of TEPs and the requirements of the Na/K pump

When epithelial cells form monolayer with tight junctions between cells, they separate the apical side from the basolateral side. MDCK cells in our culture started to build up TEP and TER ∼2 days in culture. The TEP and TER increased in the following 2 days, then decreased to a stable plateau in another 2 days (Fig. 1A). This is similar to the establishment of TEP across skin epidermis, which gradually increased 3–4 days after wounding and reached the peak on day 6–8 of 25 mV, then decreased to a plateau of 10 mV [8]. The exact time course may depend on the size of the wound. The rising phase, peak, and the following decrease and plateau phases are consistent. The requirement for Na^+^ transport and asymmetric distribution are the same. Our results demonstrate requirements for both Na/K pump function and asymmetry. The maintenance of a TEP is necessary for many biological processes, including transepithelial ion transport, disease diagnosis, and evaluation of the efficacies of drugs and therapies [45]–[50].

### Synchronization modulation increases TEP

Previous studies demonstrated that stimulation by a train of squared pulses increase the pump current of skeletal muscle fibers [27], [29]. We further increased the field oscillating frequency in a stepwise pattern and found the stepwise up-regulation of the pump current [23]. These results suggested that the Na/K pump can be synchronized to work at the same pace and that changing the frequency of oscillating electric fields (EFs) can modulate this pace. As a result, the function of Na/K pump can be regulated by the oscillating EFs. The direct evidence is that the activation of the pump by this synchronization modulation hyperpolarizes the resting membrane potential of skeletal muscle fibers [23], [27], [29] and cardiomyocytes [26], [30].

In the present study, we applied this technique to increase the TEP of non-excitable epithelium (Fig. 2). This merits a useful conversion of electrical energy into biological energy—a transformation of readily available energy sources into one that is scarce yet vital among living systems. This response suggests the possibility of using an external stimulus to bring forth important cellular changes.

Repeated stimulation induced repeated increases in TEP. Recovery of TEP to resting values after each round of stimulation suggests that increased TEP is not an electrical artifact of the stimulator, but rather an actual physiological response. More importantly, application of the stimulator when set to a non-modulating mode caused no increase in TEP. The underlying principle of our project is based on the idea of synchronizing the Na/K pumps to work at the same pace and then modulating them with an increasing oscillation frequency to maximize their working capacity. Thus, it follows that synchronization without modulation will not increase the TEP, which is indeed what we observed when we applied the stimulator at a constant or decreasing oscillating frequency.

### Synchronization modulation on Na/K pump

The Na/K pump, also known as the Na/K-ATPase, plays an essential role in cells' functions. The pump activity maintains transmembrane ionic gradients and cell membrane potentials that regulate cell volumes and generate driving forces to secondary active transport of other substances across the cell membrane. The voltage-dependent activation of this molecule has been extensively studied [29], [51]–[53]. Various electric fields (EFs) were also applied to stimulate the pump function [54]–[60]. An example is the application of high frequency (kHz and MHz) oscillating electric field to the pump molecules based on the hypothesis of resonance [59]. The mechanisms involved in synchronization modulation are different from those studies. We are first entraining the pump molecules to run at the same pace at the physiological turnover rate, and then gradually modulate their pumping rate.

The technique was developed on skeletal muscle fibers using whole cell patch clamp techniques. Later, the technique was applied to other excitable cells such as single cardiac cells [30], slices of cardiomyocytes [61], and smooth muscle cells of peripheral blood vessels [62]. The results showed that on these excitable cells, the synchronization modulation technique can effectively activate the pump function. In this study of monolayer of epithelial cells three types of independent experiments confirmed that SM stimulation target the Na/K pump. First, when the pump is blocked by pharmacological agents, the increase in TEP by SM stimulation is partly or completely abolished. They selectively inhibit the Na/K pump by stabilizing it in the 3Na^+^-binding E2-P transition state so that sodium cannot be extruded to the extracellular side in which the pump remains open to [35].

Second, the increase in TEP induced by the stimulation requires asymmetric localization of Na/K pump. We used butyrate, a four-carbon fatty acid known to disrupt delivery of Na/K pump to the basolateral surface of MDCK cells. After butyrate treatment, two subunits of the Na/K pump are inappropriately delivered to the apical surface instead of the basolateral surface [37]. Treatment with butyrate alone significantly decreased TEP and abolished stimulation induced TEP increases. More importantly, the exclusive basolateral localization of the Na/K pump in MDCK epithelium enables a net accumulation of positive charges on one side [37], [63]. This layout directly contributes to an increase in TEP upon stimulation, and consequently, a dissipation of that gradient when ouabain or digoxin is added to block the Na/K pump.

Again we exploit the concept of a net positive charge accumulation on the basolateral side due to unidirectional localization of the Na/K pump. If there were a decrease in pump activity, as in butyrate treatment, there would be an overall drop in basolateral concentrations of positive ions. Furthermore, if there were bidirectional targeting of the pump, the appearance of positive charges on the apical side counterbalances those on the basolateral side, leading to a relative decline in basolateral to apical transepithelial positive gradient. Because these two mechanisms clearly blunt the Na/K pumps' effects, we posit that they likely contribute to the striking changes seen with butyrate treatment. Junctional disruption alone cannot explain this phenomenon because butyrate-treated cells still maintained a relatively high TER of 2.7 KΩ×cm^2^ but showed minimal TEP response to SM compared with normal cells undergoing re-stimulation (Fig. 2B & C), which have a much lower TER of<1 KΩ×cm^2^ but still responded well to stimulation.

We noticed an immediate decrease in TER after all SM stimulations. This is likely due to possible side effects of an increase in conductance induced by the stimulation. Even though with the resistance reduced, SM stimulation could still increase TEP (Fig. 2B). This suggests that optimized stimulation may reduce the side effects and further increase the TEP more efficiently.

### Physiological relevance

The Na/K pump is not only important in the regulation of transmembrane potentials, but is also essential in vectorial transepithelial ion transport [64]. The Na/K pump catalyzes ATP and provides the energy source for directional transport of Na^+^/K^+^. The directional transport of ions together with the electrical resistive barriers formed by tight junctions generates and maintains the TEP. TEP occurs in many types of epithelia such as the skin, cornea, airway, kidney, gastrointestinal tract, and prostate, and it is the mechanistic basis for the generation of wound EFs [17], [26]. Inhibition of the Na/K pump by ouabain reduces TEP [65]–[66]. Pharmacological manipulations of TEP, by either increasing or collapsing TEP, affect the corneal wound healing rates by enhancing or decreasing the wound EFs [68], [69]. Because wound EFs have been considered to be an overriding guidance cue in wound healing [17], regulation of TEP could contribute to the wound healing process by controlling wound EF strength. Our measured absence of a TEP after making a linear wound to MDCK monolayers sets the stage for potentially increasing TEP to enhance wound healing.

In conclusion, we demonstrated that (1) synchronized modulation of the Na/K pump activity is not limited to a single cell—the established technique and principles also apply to a monolayer of epithelium. (2) The mechanisms underlying synchronized modulation could be similar to that in the single cell, but it largely relies on the tight junction, Na/K pump distribution, and cell type of the epithelium. (3) Increase in TEP may be implicated in wound healing, because the increase in TEP could enhance the wound electrical fields that may enhance wound healing.
